# Charting a Course for Leader and Leadership Education and Development in American Medical Schools

**DOI:** 10.15694/mep.2018.0000037.1

**Published:** 2018-02-14

**Authors:** Neil E. Grunberg, Erin S. Barry, Hannah G. Kleber, John E. McManigle, Eric B. Schoomaker

**Affiliations:** 1 Uniformed Services University of the Health Sciences

**Keywords:** Leadership Education and Development, Leaders, Leadership, Medical Leadership Education and Development, LEAD

## Abstract

This article was migrated. The article was marked as recommended.

**Problem**: Leadership has been identified as an essential component for success in medicine. Many medical schools have initiated Leader and Leadership Education and Development (LEAD) programs to develop physician leaders. Currently, there is no consensus whether teaching leadership is important, who to teach, what topics to teach, and where leadership fits into the curriculum during medical school.

**Approach**: To address these issues, the Uniformed Services University of the Health Sciences (USU) LEAD team convened an inaugural Medical Student LEAD Summit and Working Group Meeting on April 4, 2017. Participants came from public and private U.S. medical schools engaged in LEAD programs, military service academies, the Veterans Administration, and the Association of American Medical Colleges. The purpose of this meeting was to share opinions, experiences, and current practices regarding medical student LEAD.

**Outcomes**: Participants overwhelmingly agreed that: (1) providing LEAD is an essential component of undergraduate medical education; (2) there currently is no single best LEAD program for all medical schools; (3) a clear purpose, goal, philosophy, and conceptual framework consistent with the mission and vision of each institution is needed; (4) assessment of students, programs, faculty must be incorporated; and (5) research and scholarship are essential for LEAD programs.

**Next Steps**: Based on the positive feedback and interest from participants, the USU LEAD team will host a second Summit in April 2018 to follow up with the inaugural participants and to include representatives from additional institutions who are currently conducting or interested in starting their own medical school LEAD programs.

## Problem

The Association of American Medical Colleges (AAMC) lists leadership as an essential component for success in medicine. In recent years, several medical schools have added Leader and Leadership Education and Development (LEAD) programs to their curriculum. Currently there is no consensus about whether to require LEAD curricula in undergraduate medical education and, if so, who to teach (i.e., some vs. all students), what topics to teach, where leadership best fits into the curriculum. As the “leadership academy” of the Military Health System, the Uniformed Services University of the Health Sciences (USU) has been tasked with developing medical students as leaders since its inception. To strengthen, invigorate, and enhance LEAD programs, USU hosted a meeting for medical schools and service academies to share approaches to leader and leadership education. This paper summarizes the purpose and outcomes of a meeting that was held to share approaches and curriculum relevant to leader and leadership education in medical schools.

## Approach

The Inaugural Medical Student Leader and Leadership Education and Development (LEAD) Summit and Working Group Meeting took place on April 4, 2017, at the USU’s campus in Bethesda, Maryland. The purpose of this meeting was to share opinions, experiences, and current practices regarding medical student LEAD. The short-term goal was to create a network of professionals interested in medical student LEAD. The long-term goals were to share concepts, curricula, and programs, and to identify and develop best practices for medical student LEAD through collaboration and scholarly activities.

Participants included individuals actively engaged in leadership education. Most participants came from public and private U.S. medical schools engaged in LEAD programs for medical students. Because the military service academies emphasize leadership education, educators from the United States Air Force Academy (USAFA), United States Military Academy (USMA), and United States Naval Academy participated. Representatives from the Veterans Administration (VA) and the Association of American Medical Colleges (AAMC) also participated. See Table 1 for a list of participants.

**Table 1.** List of participants and their affiliations

Participants	Institution
Anita Navarro, EdD	Association of American Medical Colleges
Brian Clyne, MD	Brown University
Nathan Hudepohl, MD, MS, MPH	Brown University
Joe Doty, PhD	Duke University
Dean Taylor, MD	Duke University
Kathleen Calabrese, MD	George Washington University
Terry Kind, MD	George Washington University
Michael O’Leary, PhD	Georgetown University
Stephen Bohan, MD	Harvard University
Nancy Hueppchen, MD, MSc	John Hopkins University
Mark Warner, MD	Mayo Clinic
Kristi-Jo Tutela-Dane, JD	New York University
Jennie Lou, MD, MSc	Nova Southeastern University
Dean Winslow, MD	Stanford University
Erin Barry, MS	Uniformed Services University
Neil Grunberg, PhD	Uniformed Services University
Joshua Hartzell, MD, FACP, FIDSA	Uniformed Services University
Hannah Kleber, BS	Uniformed Services University
John McManigle, MD	Uniformed Services University
Francis O’Connor, MD, MPH	Uniformed Services University
Eric Schoomaker, MD, PhD	Uniformed Services University
Matthew D’Angelo, CRNA, DNP	Uniformed Services University; Graduate School of Nursing
Diane Seibert, PhD	Uniformed Services University; Graduate School of Nursing
James Dobbs, PhD	United States Air Force Academy
Matthew Clark, PhD, PMP	United States Military Academy
Melinda Kalainoff, PhD	United States Military Academy
Melanie Dodge, MBA	United States Military Academy - Preparatory School
Arthur Gibb, III, PhD	United States Naval Academy
Charles Callahan, DO	University of Maryland
David Fessell, MD	University of Michigan
Joann Quinn, PhD	University of South Florida
Suzanne Templer, DO	University of South Florida - Lehigh Valley
Ron Massey, MPA	Veterans Affairs
Angela Yarnell, PhD	Walter Reed Army Institute of Research

The meeting began with a brief plenary session to explain the background, purpose, and structure of the meeting followed by working group sessions and group discussions. For the first working group session, participants were divided into five groups based on their preferences indicated prior to the meeting and to have groups composed of participants from different institutions. The five topics for the working groups were: (1) curriculum content and delivery; (2) purpose, goals, philosophy, conceptual framework; (3) assessment of students, programs, and faculty; (4) research and scholarship; and (5) challenges and obstacles. After this session, participants reconvened and shared what was discussed, followed by an open discussion. A second working group was then held with participants joining the discussion group of their choice. As before, after the session, participants returned to summarize the deliberations followed by an open discussion. In addition, there was a featured discussant and panel discussion regarding specific LEAD program.

## Outcomes

### Overview

Participants overwhelmingly agreed that medical students need LEAD; it is an essential component of undergraduate medical education; and there currently is no single best LEAD program for all medical schools.

### Curriculum Content and Delivery

With regard to leadership curriculum for medical students, participants determined that there currently is no consensus about specific content and delivery, due in large part to the uniqueness of each medical school. Participants discussed the importance of life cycle leadership development (undergraduate medical education, graduate medical education, faculty development). It is important to include and distinguish education about leadership, leaders, managers, and followers; to teach leadership in pre-clerkship courses and clerkship rotations; to include individual, team-based, and experiential learning; and to incorporate real world examples and relevant issues and applications. Participants agreed that it is important to include near-peer and peer-peer education as well as experience giving and receiving feedback. It would be valuable to establish and align competencies with the entire curriculum and its goals; to include student projects; and to incorporate mentoring and coaching.

### Purpose, Goals, Philosophy, Conceptual Framework

Participants agreed that it is important for LEAD programs to have a clear purpose, goal, philosophy, and conceptual framework consistent with the mission and vision of each institution. Further, it is important to distinguish between leaders (human capital) and leadership (social capital) and to understand that students need to acquire knowledge, attitudes, and skills as leaders, managers, and followers. Discussions addressed the value of fostering professional identity as leaders (in addition to identities as physicians) and to understand the different cultures within which leaders must act.

### Assessmentsof Students, Programs, and Faculty

Assessments can measure individual leaders’ strengths and weaknesses, evaluate the program, track student progress, and evaluate faculty. Therefore, it is important to determine what, when, who, where, and how to assess the impact of leader education and leadership development programs; how best to provide feedback based on assessment information; and to collaborate across institutions to establish norms in assessment and performance. Participants agreed that assessment is important but that currently there is no consensus about leader and leadership assessment. Design of assessment cannot be done without first developing a conceptual framework and associated curriculum.

### Research and Scholarship

Participants agreed that LEAD programs should be based on sound theories, concepts, principles, and evidence. Therefore, research and scholarship should address topics discussed by the other working groups. Potential activities include: outcomes research based on program goals; determination of optimal pedagogical teaching strategies to teach about leadership; and reliable and valid assessment of knowledge, skills, and attitudes relevant to effective leadership. It also is important to incorporate scholarship that studies, evaluates, and integrates relevant information about LEAD for medical students from relevant fields including psychology, sociology, business, anthropology, as well as studies of various leader types, styles, and effectiveness.

### Challenges and Obstacles

Participants agreed that adequate resources (e.g., personnel, time, funds) are key and essential to establish and deliver meaningful LEAD programs in medical schools and that buy-in is critical from the institution, administration, faculty, and students. It is important to determine how best to train and recruit appropriate teachers and facilitators; to use experiential applications and involve project work; to recognize that time to teach leadership is limited and, therefore, must be optimally effective and time-efficient.

### Featured Discussant

Between the working group/plenary discussion sessions, Dr. Michael O’Leary (Faculty member, Georgetown University McDonough School of Business and Presidential Leadership Scholars Program) spoke to the group about big picture ideas regarding how to build successful LEAD programs. Dr. O’Leary indicated a need to consider identity, inspiration, ability, and approach. It is important for students to understand who they are and know their knowledge base (Identity). He suggested that students should develop “muscle memory” with regard to leadership to be learned via relevant examples and experiential learning that is flexible and tailored to their needs to get them to understand why it is important and emulate good examples (Inspiration). Students need to understand what they need to know now and educators must remember that students do not need to know everything now (Ability). He suggested that educators focus more deeply, with more flexibility, and with meaningful, “at scale” examples (Approach). This approach meets students at their level and teaches them in the best way possible, to include using technology and peer/near-peer students as co-teachers wherever possible.

### Panel Discussion Regarding Specific LEAD Programs

Representatives from five schools made brief presentations describing their LEAD programs. Key points from each presentation are listed below.

**Brown University.** Brown School of Medicine (SOM) selects student groups for their leadership program which consists of 15, two-hour sessions that include leadership interviews with faculty to get advice and reflect; case-based discussions; leadership action session projects; and student presentations to community leaders (field work with real connections). This program focuses on general and targeted needs assessment (e.g., what would a 2 ^nd^ year student on the wards look like and teach those skills that are needed; https://vivo.brown.edu/display/bclyne).

**University of Michigan.** University of Michigan SOM’s program includes required and optional components throughout the four years of education for all students. These components include life coaching sessions in year 1, conversations with leaders throughout the year, and sessions in which students discuss their core challenges in life (facilitators also share their stories). There is a leadership tool that students can use to track their experiences with prompts for self-reflection about their experiences and improvisation sessions dealing with hypothetical cases ( https://medicine.umich.edu/medschool/education/md-program/curriculum/longitudinal-learning/leadership-program).

**Duke University.** The Feagin Leadership Program (24 Scholars/year in four, 6-person teams) is a nine-month leadership immersion program for 3 ^rd^ year students, MD-PhD students, and residents and fellows from all specialties. It involves competitive selection to participate; didactic sessions, individual coaches, team experiences, mentoring, peer-peer learning; leadership coaches; and a capstone event for participants to present their team projects. Duke has created a Healthcare Leadership Model (focused on Patient-Centeredness) and developed a LEAD Curriculum that is threaded through all four years of medical school. The curriculum is based on the Healthcare Leadership Model (emotional intelligence, teamwork, selfless service, integrity, critical thinking), includes near-peer learning, and provides opportunity to pursue a Certificate in Leadership Development ( https://www.feaginleadership.org/).

**University of South Florida.** The USF SOM Scholarly Excellence Leadership Experiences Collaborative Training (SELECT) program educates 50 students/year. Students complete assessments of emotional intelligence, empathy, burnout, conflict, and personality for professional development. The program involves coaching sessions (8 students with 2 faculty) with the same coaches for all four years; comprehensive exams (one formative, one summative) each year; and a summer immersion course. During their 3 ^rd^ year clinical rotations held at Lehigh Valley, PA, students engage in 1-on-1 sessions with coaching faculty to discuss plans to be more successful, class topics, and developing a professional development plan ( http://health.usf.edu/medicine/mdprogram/select).

**Uniformed Services University.** USU’s LEAD program is required for all medical students and is provided across all four years. It includes plenary sessions, small groups, and applications in medical field settings based on the FourCe-PITO conceptual framework that includes: Character, Competence, Context, Communication across four levels of psychosocial interaction - Personal, Interpersonal, Team, Organizational. Topics include: principles and types of leadership; personality; emotional and social intelligence; effective communication; leaders, managers, followers; team building; self and peer assessment; and performance under stress. There are optional leadership capstone projects for 3 ^rd^ and 4 ^th^ year medical students and ongoing scholarly projects regarding relevant concepts and assessment ( https://www.usuhs.edu/usulead).

## Next steps

After the Summit, a survey was sent to participants regarding reactions and suggestions for future sessions. Respondents suggested the creation of a shared, electronic, resource platform to post ideas and curricula. Respondents also indicated a desire for follow-up meetings to include: annual meetings; each of the five topics focused upon separately; or a focus on curriculum, details about existing programs, and assessment. Interest also was expressed to continue networking and to engage in collaborative studies and program development.

Based on the success and interest of this inaugural meeting, the USU LEAD team will host a Summit in April 2018 to follow up with the inaugural participants and to include representatives from additional institutions who are conducting or interested in starting medical school LEAD programs. The 2018 meeting will focus on curriculum and assessment.

## Take Home Messages

• Medical Education should include leadership education and development
• There currently is no single best established curriculum for medical leadership education
• It is important to consider: (1) curriculum content and delivery; (2) purpose, goals, philosophy, conceptual framework; (3) assessment of students, programs, and faculty; (4) research and scholarship; and (5) challenges and obstacles
• Working group meetings focused on medical leadership education and development are valuable

## Notes On Contributors

***Neil E. Grunberg***, PhD, is a Professor in the Department of Military & Emergency Medicine (MEM) and Director of Research & Development of Leader and Leadership Education and Development (LEAD), Uniformed Services University of the Health Sciences (USU).

***Erin S. Barry***, MS, is a Research Assistant Professor in MEM, Research Associate for LEAD, and doctoral student in the Health Professions Education Program, USU.

***Hannah G. Kleber***, BA, is an Education Specialist for LEAD, USU.

***John E. McManigle***, MD, is Deputy Dean & Senior Advisor in MEM and Director of Curriculum of LEAD, USU.

***Eric B. Schoomaker***, MD, PhD, is a Professor and Vice Chair for Leadership, Centers & Programs in MEM and Director of LEAD, USU.

