# Supplementary material for: Charting a Course for Leader and Leadership Education and Development in American Medical Schools
Source: MedEdPublish (2016). 2018 Feb 14;7:37. [Version 1] doi: 10.15694/mep.2018.0000037.1 (PMC10711938; doi:10.15694/mep.2018.0000037.1)
Supplement: Peer review history

# Reviewer report: Reviewer response for version 1

**DOI:** 10.21956/mep.19514.r28867

**Published:** 2018-02-16

**Authors:** P Ravi Shankar

**Affiliations:** 1 American International Medical University

This review has been migrated. The reviewer awarded 4 stars out of 5 Leadership education is important for medical students and doctors. The paper describes an Inaugural Medical Student Leader and Leadership Education and Development (LEAD) Summit and Working Group Meeting held in April 2017 in the United States (US). The meetings are planned to be an annual affair with the next one scheduled for April 2018. I agree with the other reviewers about the focus of the paper being US-centric but the authors have clearly mentioned this in the title of their paper which provides readers an idea of what to expect from the paper. As mentioned by the other reviewers the authors have not placed their manuscript within the context of current literature on the subject and no references have been provided. This manuscript is a description of a new educational method or tool and more details about leadership education implementation can be provided. The examples of programs at specific medical schools with links to the descriptions on the website will be useful. Leadership is an interdisciplinary area and I am interested in knowing which combination/group of faculty members are best suited to facilitate learning of the subject. Also the areas of leadership which are most important and relevant may vary between countries, regions and will also vary according to the type of institution or level of care provided. For many developing countries primary health care is vitally important and most doctors are trained for their role as leaders of a primary health care facility. Similar to what has been done in the US other countries could conduct a similar meeting to address the leadership curriculum issues of importance in their setting.

Reviewer Expertise:

NA

No decision status is available

# Reviewer report: Reviewer response for version 1

**DOI:** 10.21956/mep.19514.r28869

**Published:** 2018-02-15

**Authors:** Gert Van Zyl

**Affiliations:** 1 University of the Free State

This review has been migrated. The reviewer awarded 4 stars out of 5 Well written paper and to the point with confirmation of a number of facts on leadership and leadership education. A piece of research faculties can use in their undergraduate programmes but also link up with faculty development plans for senior academics and also to prepare staff identified for succession planning. The importance of the concept of mentoring and coaching on undergraduate level, but also on higher levels, is confirmed.

Reviewer Expertise:

NA

No decision status is available

# Reviewer report: Reviewer response for version 1

**DOI:** 10.21956/mep.19514.r28866

**Published:** 2018-02-15

**Authors:** Judy McKimm

**Affiliations:** 1 Swansea University

This review has been migrated. The reviewer awarded 3 stars out of 5 This paper provides a useful description of a leadership summit and introduces some case studies of leadership development programmes in medical education. I think this is an interesting introduction for those who are new to Leadership development however the paper is unreferenced and as such, does not locate the summit and case examples within the international literature on leadership development at both undergraduate and postgraduate levels.I think it would be a nice follow up to explore international examples ( such as the UK Medical Leadership competency framework) and work being carried out to develop a ‘core curriculum ‘ in Leadership for undergraduates. It would also be good to locate this work within the Leadership literature and to clearly distinguish and define terms (Leadership, Management and followership) as these can be contested. However, I found this a really good summary of work going on in the US in Leadership development.

Reviewer Expertise:

NA

No decision status is available

# Reviewer report: Reviewer response for version 1

**DOI:** 10.21956/mep.19514.r28868

**Published:** 2018-02-14

**Authors:** Johnny Lyon Maris

**Affiliations:** 1 Southampton GP Education Unit

This review has been migrated. The reviewer awarded 4 stars out of 5 Leadership in education and medicine is an important and under-explored curriculum area. The authors describe leadership programmes in their institutions and sum up key areas. The article is well written as a description of an intercollegiate committee and comparison of leadership programmes, It is useful for other academic institutions who wish to review their curriculum.

Reviewer Expertise:

NA

No decision status is available

# Reviewer report: Reviewer response for version 1

**DOI:** 10.21956/mep.19514.r28870

**Published:** 2018-02-14

**Authors:** Trevor Gibbs

**Affiliations:** 1 AMEE

This review has been migrated. The reviewer awarded 4 stars out of 5 Although this paper is rather US-centric, I think that there is a lot to be learned by others. I am not sure that the findings are that unusual but I like the way that the summit has brought together some very important elements / steps within curriculum leadership. I liked the examples provided by some of the schools involved. All in all I feel that its apparent simplicity makes it an interesting and valuable paperI think that this is a useful paper for those involved in the area of leadership education and development and I look forward to see how this group develops and hopefully in their dissemination of their work together.

Reviewer Expertise:

NA

No decision status is available
